# Macro-to-Micro Structural Proteomics: Native Source Proteins for High-Throughput Crystallization

**DOI:** 10.1371/journal.pone.0032498

**Published:** 2012-02-29

**Authors:** Monica Totir, Nathaniel Echols, Max Nanao, Christine L. Gee, Alisa Moskaleva, Scott Gradia, Anthony T. Iavarone, James M. Berger, Andrew P. May, Chloe Zubieta, Tom Alber

**Affiliations:** 1 Department of Molecular and Cell Biology, University of California, Berkeley, California, United States of America; 2 European Molecular Biology Laboratory, Grenoble, France; 3 QB3 Institute, Berkeley, California, United States of America; 4 Fluidigm Corporation, South San Francisco, California, United States of America; German Cancer Research Center, Germany

## Abstract

Structural biology and structural genomics projects routinely rely on recombinantly expressed proteins, but many proteins and complexes are difficult to obtain by this approach. We investigated native source proteins for high-throughput protein crystallography applications. The *Escherichia coli* proteome was fractionated, purified, crystallized, and structurally characterized. Macro-scale fermentation and fractionation were used to subdivide the soluble proteome into 408 unique fractions of which 295 fractions yielded crystals in microfluidic crystallization chips. Of the 295 crystals, 152 were selected for optimization, diffraction screening, and data collection. Twenty-three structures were determined, four of which were novel. This study demonstrates the utility of native source proteins for high-throughput crystallography.

## Introduction

Since the advent of recombinant DNA technology, structural and biochemical research has focused increasingly on the characterization of recombinantly expressed prokaryotic and eukaryotic proteins. However, statistics from the National Institutes of Health Protein Structure Initiative (PSI) (http://targetdb.pdb.org/statistics/TargetStatistics.html) indicate that of successfully clones and expressed proteins, less than 5% of the targets selected result in a crystal structure. Protein production, purification, and crystallization remain a series of bottlenecks for large scale structural studies of any given genome [1]. These results suggest that new approaches are warranted in order to access the great majority of proteins and protein complexes that cannot be facilely recombinantly expressed for structural and biochemical analysis.

To fill this gap, we investigated the feasibility of native-source protein purification as part of a high-throughput crystallization and structure determination pipeline. The methodologies described provide a complementary approach to current structural genomics initiatives. By providing an alternative to recombinant technology for protein production, the native source purification and crystallization pipeline outlined here can potentially expand the scope of structural studies to proteins that currently cannot be obtained or are difficult to obtain by recombinant DNA techniques due to low levels of expression, poor solubility, the lack of necessary post-translational modifications, or instability due to missing partners in the native protein complex. Based on these experiments, we demonstrate successful structural characterization of multiple proteins using only microgram quantities of purified material. By scaling up the amount of starting material and introducing atypical methods of filtration and fractionation, we obtained sufficient quantities of 408 unique samples for crystallization trials. Simultaneously, scaling down the amount of protein sample used for crystallization, enabled structure determination of protein species from native sources.

## Results

*Escherichia coli* was chosen as a model system in this study due to its relatively small and structurally well-studied proteome and fully-sequenced genome [2], [3]. Of the ∼4243 predicted ORFs in the *E. coli* proteome, over a quarter are likely to encode membrane associated or membrane bound proteins. This study focused on the soluble portion of the proteome. In a typical experiment, large-scale fermentation (120 L) was used to provide sufficient starting material for downstream purification and crystallization. To maximize access to soluble proteins, we grew the cells aerobically to log phase at 37°C in minimal media. Large scale fermentation allowed the production of kilogram quantities of cells, while minimizing the deleterious effect of high cell density on protein quality. Automated fermentation was necessary to monitor the growth conditions, maintain appropriate aeration, control pH, and to produce enough starting material for downstream crystallization experiments [4].

**Figure 1:**
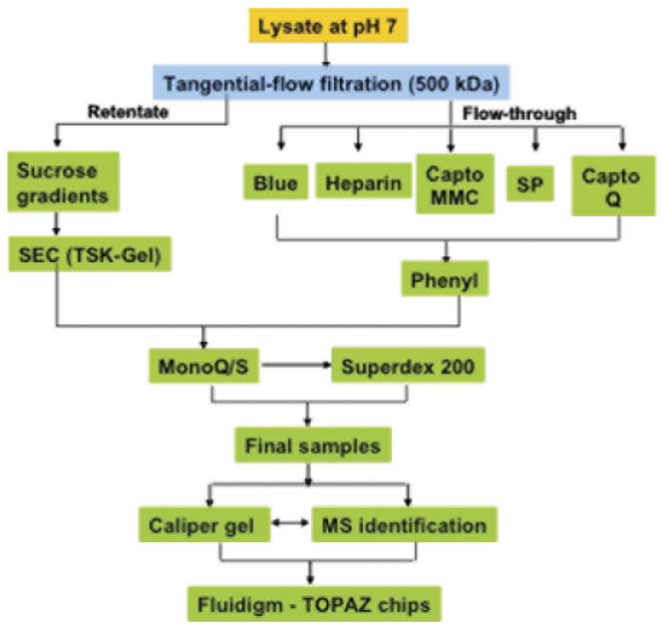
Proteome fractionation and purification flow chart. Approximately 500 g of *E. coli* cells were lysed at pH 7 using a microfluidizer and the cell debris pelleted. The supernatant was applied to a tangential flow column with a nominal molecular weight cut off of 500 kDa, generating 2 fractions (retentate and flow through). The fraction above 500 kDa (retentate) was further purified via sucrose gradients, size exclusion, and ion exchange chromatography prior to crystallization trials. The fraction less than 500 kDa was applied to multiple affinity and ion exchange columns followed by phenyl sepharose, ion exchange, and size exclusion prior to crystallization trials in microfluidic chips.

**Figure 2:**
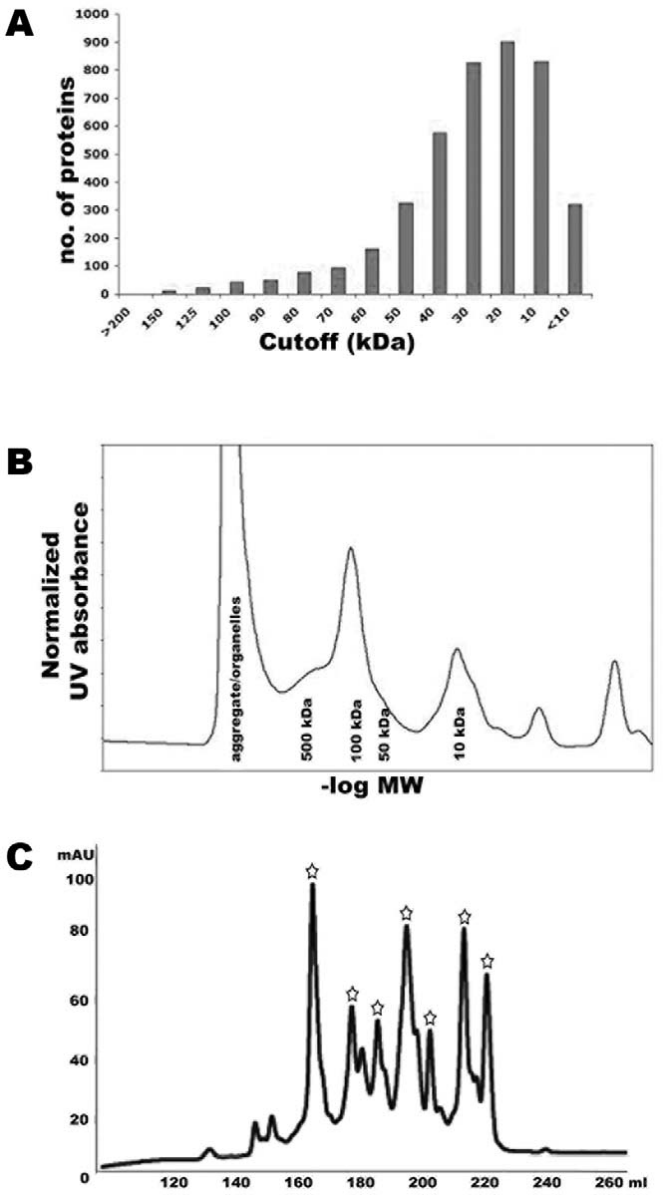
*E. coli* proteome predicted and experimental characterization. (A) Predicted size distribution of all ORFs in the *E. coli* proteome. (B) Size exclusion chromatograph of crude *E. coli* lysate with the largest peak at approximately 100 kDa. (C) Final step ion exchange (MonoQ) purification in a typical fractionation experiment. Peaks marked with a star were sent for downstream crystallization trials.

Purification of proteins from a native source presented very different challenges compared to recombinantly overexpressed and affinity tagged proteins. To successfully purify unique protein samples from the native proteome, a series of orthogonal steps were used (Figure 1). Initial fractionation steps relied on rapid tangential flow methods and pilot-scale ion exchange chromatography using new high-capacity resins to process large amounts of *E. coli* lysate (0.5–1 kg cells). Based on size predictions of all predicted ORFs present in *E. coli*, a large peak at approximately 20 kDa was expected from initial profiling of the proteome (Figure 2A). The soluble proteins, however, had large peaks at void volume (over 500 kDa) and approximately 100 kDa (Figure 2B), suggesting that many proteins were either aggregated or forming large multimeric complexes. In order to separate these two peaks, an initial step of tangential flow filtration was used to allow the rapid generation of a rough cut between a high molecular weight fraction (over 500 kDa), and a lower molecular weight fraction. While the higher molecular weight fraction likely contained aggregates, organelles, and soluble protein complexes, this fraction was not extensively pursued. Further fractionation using sucrose gradients was attempted; however no crystals were obtained from the high molecular weight fraction leading us to focus on the fraction under 500 kDa.

The <500 kDa fraction was further purified through a series of orthogonal steps including the first ion exchange step on pilot-scale columns with step elution at salt concentrations ranging from 0.01 to 1 M. Ion exchange allowed the selection of different pools of proteins based on the isoelectric point (pI) and enabled initial proteome simplification to create reproducible and manageable subsets of proteins. The proteome subsets were subjected to a series of downstream purifications including affinity purification, hydrophobic interaction chromatography, gel exclusion, and high-resolution ion exchange chromatography. Final fraction purity ranged from approximately 95% to less than 5%, with the majority of fractions comprising at least 30% of one protein species (Figure 2C). By fractionating the soluble portion of the proteome and retaining all fractions for further subdivision in sequential steps, the number of unique samples for downstream crystallization was maximized from a single preparation of bacterial cells. In addition, as the number of fractions increased exponentially during purification, fractions were frozen to facilitate handling and processing. By retaining all fractions during purification, the number of fractions processed to final purity could be easily scaled depending on available time and personnel. The large scale production and purification of proteins from the soluble *E. coli* proteome constituted a macro scale step in the crystallization pipeline. To our knowledge, this represents the first use of pilot systems for whole-proteome fractionation and subsequent crystallization. The proteins produced from this step were then subjected to microscale characterization and crystallization using microfluidic technology.

To maximize the number of protein crystal structures solved, the crystallization platform needed to effectively use the small amounts of available samples from native purification. Microfluidic crystallization allowed routine sampling of 96 conditions with as little as 10 µg of protein [5], [6], [7]. Scaling up protein production and purification yielded enough material for thousands of individual crystallization trials, even for samples present in relatively low abundance (less than 100 ug of purified protein). Thus, even a small amount of sample was sufficient in many cases to screen and optimize crystals for X-ray structure determination at a synchrotron source.

**Figure 3:**
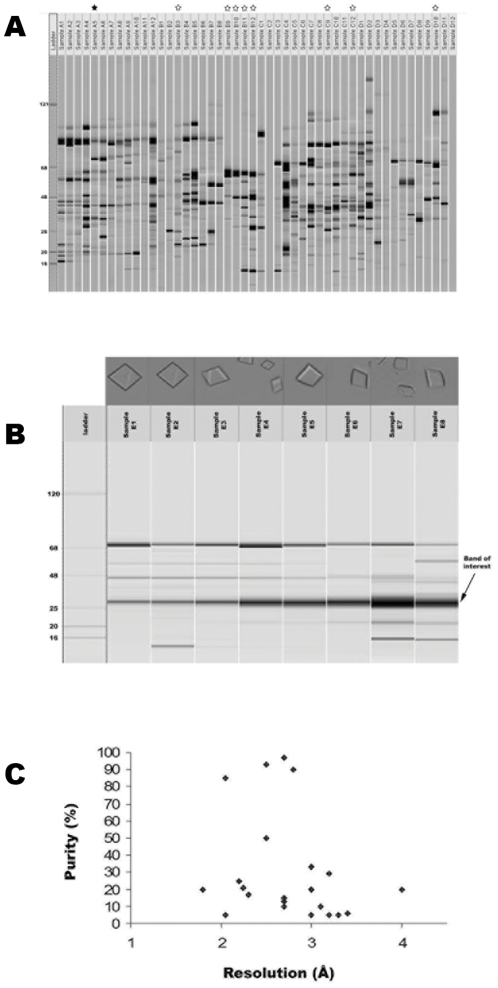
Crystallization of native source *E. coli* proteins. (A) Capillary electrophoresis of purified protein fractions. White stars indicate samples successfully crystallized and black stars represent solved structures. (B) Crystals of 5-keto-4-deoxyuronate isomerase crystallized from fractions of varying purity. Crystal quality was not always correlated with sample purity. (C) Resolution of the data collected versus percent purity of the starting sample based on quantification of protein concentrations by capillary gel electrophoresis with the Caliper system. Sample purity did not correlate with higher resolution data.

The proteome fractionation step in the pipeline yielded 408 unique fractions, as identified by capillary electrophoresis, containing one or more proteins (Figure 3A). All these fractions were used in crystallization trials in microfluidic chips. Surprisingly, sample complexity did not correlate with crystallization hits or crystal quality (Figure 3B and C). We obtained crystals in 295 of the 408 fractions, representing 73% of the total fractions obtained. Out of these, we focused on 152 of the higher quality crystals, where crystal quality was scored based on size and morphology. Of the fractions that were selected for crystal optimization and data collection, 37 unique datasets were collected at synchrotron beamlines, with a maximum resolution ranging from 1.8 Å to 7 Å. Twenty eight of the 37 datasets had a resolution better than 3.5 Å.

Protein identification was done after successful crystallization and x-ray data collection. Due to limited available sample and the high complexity of many of the crystallized fractions, mass spectrometry was only successful in identifying approximately 20% of the crystallized proteins. Brute force molecular replacement trials were used to identify proteins in the majority of the crystallized and well diffracting samples. In ∼80% of the cases tested here, where the resolution was 3.5 Å or better, this strategy was successful in identifying the protein and solving the crystal structure (Table 1). Brute-force molecular replacement trials were conducted using 10,747 structures in the PDB with at least 30% sequence identity to an *E. coli* ORF. Each data set collected was screened against all search models using the program MOLREP [8]. Rotation function Z-scores were calculated and an arbitrary cut-off between 6 to 7 was used to determine whether a potentially correct solution had been found prior to a full translational search using MOLREP and/or PHASER [9]. For all structures solved, visual inspection of the electron density map was sufficient to unambiguously determine whether or not the solution was correct. Only novel structures not present in the PDB were further refined.

**Table 1:** Crystallization conditions and data collection statistics for previously deposited structures.

PDB ID	Resolution this study	Resolution PDB	Rsym	I/sigI	R-free	Crystallization conditions	Protein description
1IPW	3.2	2.3	0.179	3.6	0.41	15–20% PEG 1000, 100 mM MES pH 6.0, 300–400 mM KOAc	Inorganic pyrophosphatase
1XRU	3.0	1.94	0.065	10.1	0.31	1.5 M Ammonium sulfate, 0.1 M MES, pH 6.5	5-keto-4-deoxyuronate isomerase
1N57	2.8	1.6	0.122	15.5	0.27	0.1 M MgCl2, 0.1 M Tris pH 8.5, 20% PEG 10,000, 0.5% ethyl acetate	Hsp31
1R2K	2.0	2.1	0.054	17.4	0.29	1.4 M sodium citrate tribasic dihydrate, 0.1 M Hepes, pH 7.5	MoaB (molybdopterin biosynthesis protein B)
1BJN	2.2	2.3	0.052	20.0	0.29	0.2 M MgCl2, 0.1 M HEPES pH 7.5, 15% PEG 3350	phosphoserine aminotransferase
1GG9	2.5	1.89	0.126	7.2	0.28	200 mM NaCl, 100 mM Tris pH 8.5, 20% PEG 3350	Catalase HPII
1PKY	3.2	2.5	0.08	20.5	0.34	0.05 M Ammonium sulfate, 0.05 M Bis-Tris, pH 6.5, 30% pentaerythritol ethoxylate	Pyruvate kinase I
1CS1	3.25	1.5	0.109	5.7	0.36	45% polypropylene glycol 400, 0.1 M Bis-Tris, pH 6.5	Cystathione gamma-synthase
1TJ7	3.0	2.44	0.166	21.6	0.29	0.1 M Bis-Tris pH 6.5, 1.6 to 0.8 M Ammonium sulfate.	Ariginosuccinate lyase
1BBW	3.4	2.7	0.142	9.4	0.35	0.1 M Tris pH 8.5, 25% PEG 3350, 3% isopropanol	Lysyl-tRNA synthetase (constitutive)
1NXG	3.3	2.5	0.077	8.4	0.32	0.2 M Lithium sulfate monohydrate, 25% PEG 3350, 0.1 M Tris, pH 8.5	Citrate synthase
1DHP	3.0	2.3	0.182	5.6	0.29	0.1 M HEPES pH 7.5, 10–25% PEG 3350	Dihydrodipicolinate synthase
1YAC	2.3	1.8	0.106	6.8	0.25	0.1 M Bis-Tris pH 6.5, 45% Polypropylene glycol P 400	ycaC gene product
1×12	4.0	2.0	0.129	5.3	0.42	0.2 M NaFormate, 20% PEG 3350, 3% MeOH	Nicotinamide nucleotide transhydrogenase domain I
1YE9	2.5	2.8	0.174	11.4	0.33	0.1 M MgFormate, 18% PEG 3350	Catalase HPII (truncated)
1HOT	2.2	2.4	0.134	16	0.34	0.056 M sodium phosphate monobasic monohydrate, 1.344 M potassium phosphate dibasic, pH 8.2	Glucosamine 6-phosphate deaminase
1CG1	2.7	2.5	0.051	26.6	0.35	0.1 M TRIS pH 8.5, 25% PEG 3350	Adenylosuccinate synthetase
2PWZ	1.6	2.2	0.07	27	0.42	1.1 M Sodium malonate in 0.1 M HEPES pH 7, 0.5% Jeffamine ED-2001	Malate dehydrogenase
1PMO	2.0	2.3	0.087	11.9	0.32	0.2 M Ammonium citrate tribasic pH 7, 20% PEG3350	Glutamate decarboxylase

**Figure 4:**
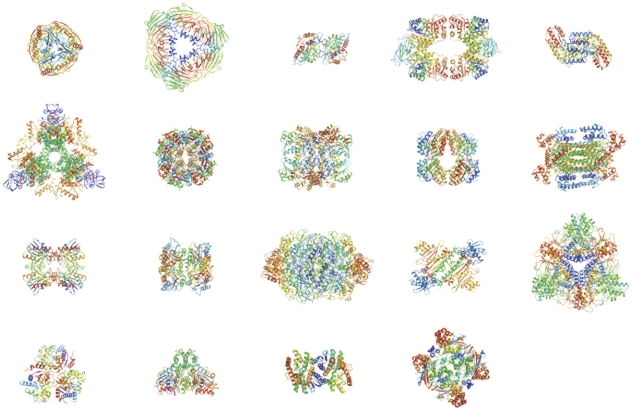
Structures of previously deposited proteins solved during the pipeline. All proteins were oligomers as shown above. Proteins are from top left - inorganic pyrophosphatase (1IPW), 5-keto-4-deoxyuronate isomerase (1XRU), Hsp31 (1N57), pyruvate kinase (1PKY), phosphoserine aminotransferase (1BJN), Citrate synthase (1NXG), ycaC gene product (1YAC), Cystathione gamma-synthase (1CS1), Dihydrodipicolinate synthase (1DHP), Arginosuccinate lyase (1TJ7), Nicotinamide nucleotide transhydrogenase domain I (1×12), MoaB (molybdopterin biosynthesis protein B) (1R2K), Catalase HPII (1GG9), Lysyl-tRNA synthetase (constitutive) (1BBW), Glutamate decarboxylase (1PMO), Glucosamine 6-phosphate deaminase (IHOT), Malate dehydrogenase (2PWZ), Adenylosuccinate synthetase (1CG1), catalase HPII truncated (1YE9).

**Figure 5:**
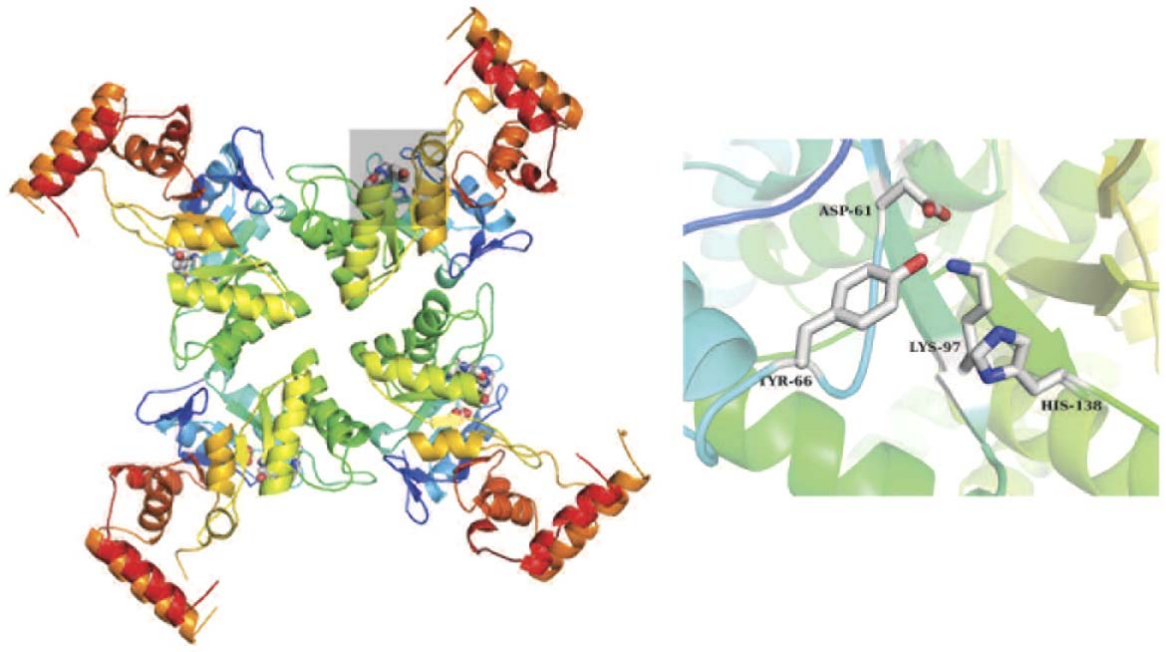
YghZ tetramer and active site. Left, the YghZ tetramer viewed along the four-fold axis. Putative active site residues are depicted as ball-and-stick and colored by atom with the active site of one monomer outlined by a gray box. Right, close -up view of the active site with putative active site residues colored by atom and labeled.

**Figure 6:**
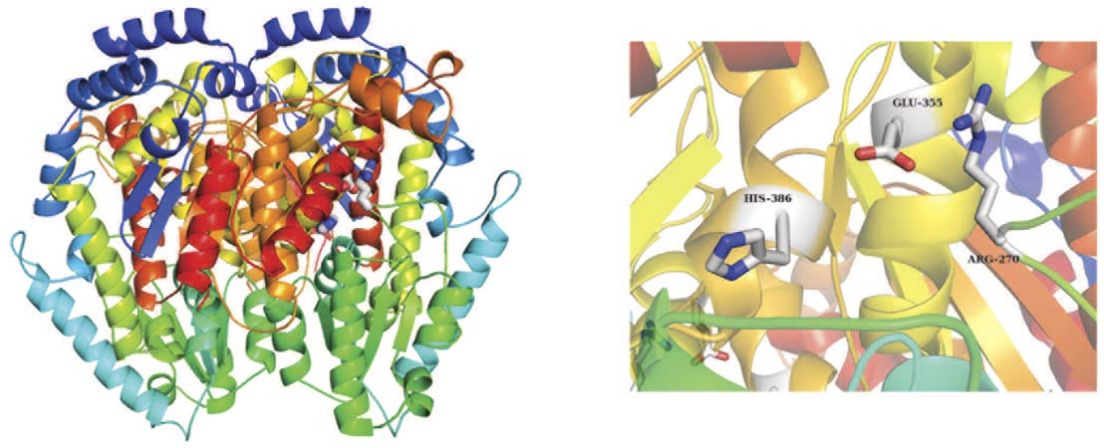
PGI dimer and putative active site. Left, the pGI dimer. Right, close -up view of the active site with putative active site residues colored by atom and labeled. The active site is formed at the dimer interface and has contributions from both monomers.

**Figure 7:**
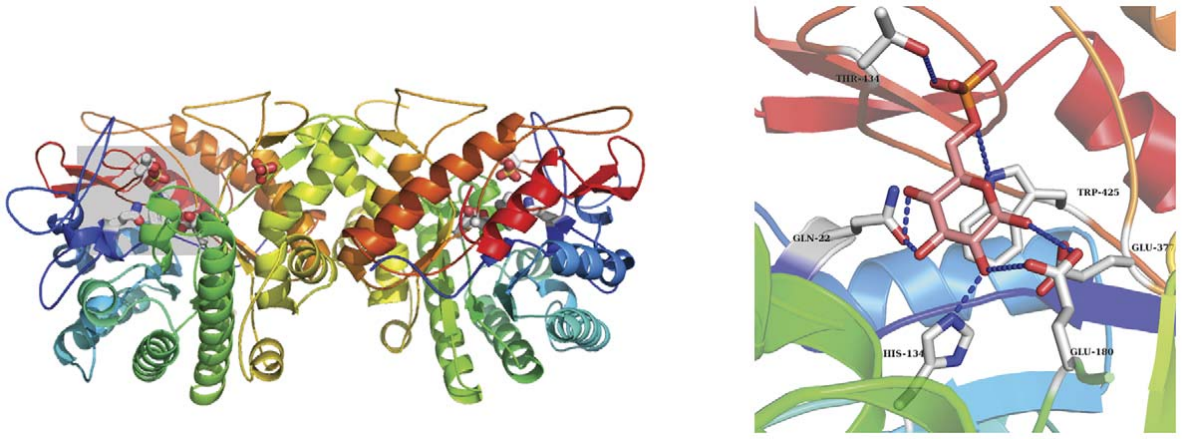
BglA dimer and putative active site. Left, BglA dimer with the putative active site outlined in a gray box. Right, close up of the active site with glucose-6-phosphate modeled based of the position of the sulfate ion from crystallization. Active site residues are depicted as ball-and-stick. Putative hydrogen bonds to the substrate are drawn as dashed lines.

**Figure 8:**
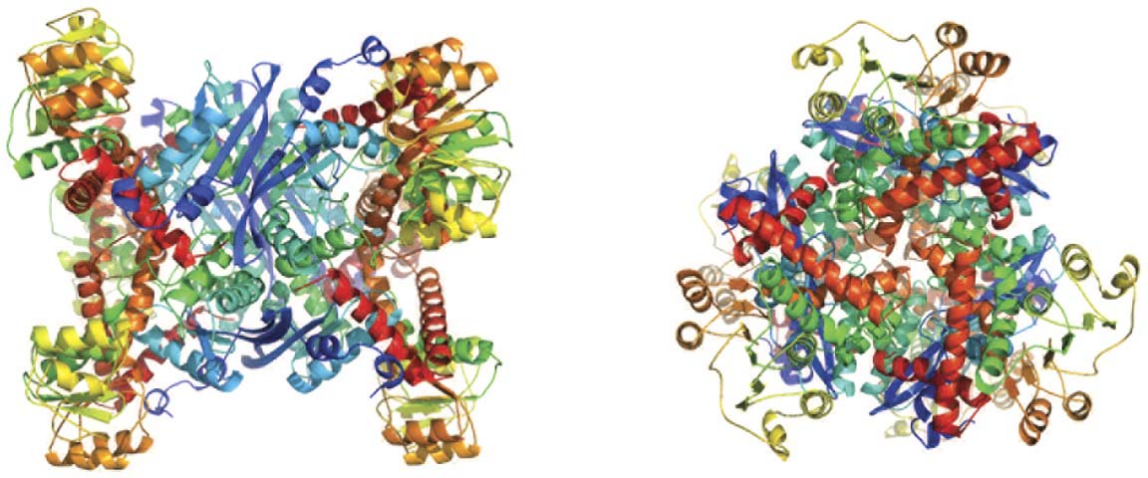
GDH hexamer from *E. coli*. The protein forms a hexamer (dimer of trimers). Left, view of the GDH hexamer along the two-fold axis. Right, view of the GDH hexamer along the three-fold axis.

In all, we determined 23 structures (Figure 4 and Table 1), four of them not deposited in the protein data bank. These novel structures represent enzymes involved in stress response, specifically methylglyoxal reductase (YghZ) (Table 2 and Figure 5), as well as three enzymes important in core metabolic functions, phosphoglucose isomerase (pGI) (Table 3 and Figure 6), 6-phospho-beta-glucosidase (BglA) (Table 4 and Figure 7), and glutamate dehydrogenase (GDH) (Table 5 and Figure 8). All the proteins are oligomeric (YghZ, pGI, BglA, GDH) and belong to three different fold families. YghZ and BglA have a classic TIM barrel structure, pGI adopts an alpha/beta fold, and GDH possesses a core Rossmann fold found in many nucleotide binding proteins. The percent identity of these four novel structures compared to deposited structures ranged from 33% (YghZ) to 65% (pGI).

**Table 2:** Data collection and refinement statistics for methylglyoxal reductase (YghZ).

	YghZ
**Data collection**	
Space group	P1
Cell dimensions	
*a*, *b*, *c* (Å)	91.70, 98.06, 98.26
α, β, ã (°)	90.3, 93.0, 106.1
Resolution (Å)	98–1.8 (1.87–1.8)
*R*_sym_	0.06(0.56)
*I*/σ*I*	9.4(1.6)
Completeness (%)	96.2 (93.3)
Redundancy	3.9(3.9)
**Refinement**	
Resolution (Å)	94–1.8
No. reflections	277925
*R*_work_/*R*_free_	17.1/20.8
No. atoms	
Protein	39762
Ligand/ion	9
Water	2644
*B*-factors	
Protein	28.0
Ligand/ion	20.0
Water	40.2
R.m.s. deviations	
Bond lengths (Å)	0.019
Bond angles (°)	1.534

*Values in parentheses are for highest-resolution shell.

**Table 3:** Data collection and refinement statistics for Glucose-6-phosphate isomerase (pGI).

	pGI
**Data collection**	
Space group	P1
Cell dimensions	
*a*, *b*, *c* (Å)	69.8, 72.9, 181.9
α, β, ã (°)	92.5, 97.8, 114.6
Resolution (Å)	179.–2.05 (2.1–2.05)
*R*_sym_	0.097(0.34)
*I*/σ*I*	7.3 (1.2)
Completeness (%)	95.0 (95.0)
Redundancy	2.0(2.1)
**Refinement**	
Resolution (Å)	89–2.05
No. reflections	186581
*R*_work_/*R*_free_	17.2/23.0
No. atoms	
Protein	25984
Ligand/ion	1
Water	3262
*B*-factors	
Protein	23.1
Ligand/ion	27.5
Water	30.3
R.m.s. deviations	
Bond lengths (Å)	0.011
Bond angles (°)	1.300

*Values in parentheses are for highest-resolution shell.

**Table 4:** Data collection and refinement statistics for 6-phospho-beta-glucosidase (BglA).

	BglA
**Data collection**	
Space group	P1
Cell dimensions	
*a*, *b*, *c* (Å)	73.7, 79.4, 98.6
α, β, ã (°)	100.0, 107.2, 102.8
Resolution (Å)	22.9–2.3 (2.42–2.3)
*R*_sym_	0.143(0.53)
*I*/σ*I*	7.9 (2.2)
Completeness (%)	92.7 (66.3)
Redundancy	4.0(3.9)
**Refinement**	
Resolution (Å)	22.9–2.3
No. reflections	82815
*R*_work_/*R*_free_	23.7/17.1
No. atoms	
Protein	16976
Ligand/ion	19
Water	1651
*B*-factors	
Protein	20.0
Ligand/ion	72.5
Water	23.78
R.m.s. deviations	
Bond lengths (Å)	0.003
Bond angles (°)	0.704

*Values in parentheses are for highest-resolution shell.

**Table 5:** Data collection and refinement statistics for glutamate dehydrogenase (GDH).

	GDH
**Data collection**	
Space group	P2_1_2_1_2_1_
Cell dimensions	
*a*, *b*, *c* (Å)	101.9 151.6 170.0
α, β, ã (°)	90.0 90.0 90.0
Resolution (Å)	40.0–3.2 (3.32–3.20)
*R*_sym_	0.164 (0.515)
*I*/σ*I*	7.9 (2.3)
Completeness (%)	99.8 (99.7)
Redundancy	3.7 (3.6)
**Refinement**	
Resolution (Å)	38–3.2
No. reflections	43835
*R*_work_/*R*_free_	0.2286/0.2653
No. atoms	
Protein	19913
Ligand/ion	8
Water	44
*B*-factors	
Protein	64.6
Ligand/ion	48.3
Water	29.5
R.m.s. deviations	
Bond lengths (Å)	0.001
Bond angles (°)	0.424

### Methylglyoxal reductase (YghZ)

The aldo-ketoreductases, of which YghZ is a member, are a large family of NADPH-dependent oxidoreductases that have the function of reducing various aldehydes and ketones [10]. The YghZ enzyme likely functions as a methylglyoxal reductase and is known to convert the toxic metabolite methylglyoxal to acetol *in vitro* and *in vivo*. As in the larger family of aldo-keto reductases, YghZ, has a central TIM barrel domain and a smaller, mostly helical domain. YghZ is a distant homolog (<40% sequence identity) of mammalian aflatoxin dialdehyde reductases of the aldo-keto reductase AKR7 family and to potassium channel β-subunits in the AKR6 family [11], [12], the structure of which was used for molecular replacement. The structure of YghZ reveals the protein forms a stable tetramer based on structural homology to related proteins in the PDB (3ERP) and the amount of buried surface area of the tetramer interface [13] (Figure 5). Based on sequence alignments and structural alignments with other aldo-keto reductases, likely catalytic residues were identified in our study. Four amino acids (Tyr-66, His-138, Lys-97 and Asp-61) form a putative catalytic tetrad in the active site. Although some extra electron density was noted in the active site, the density was too diffuse to reliably model the dinucleotide cofactor or substrate/product molecules.

### Glucose-6-phosphate isomerase (pGI)

Glucose-6-phosphate isomerase (pGI) catalyzes the reversible isomerization of D-glucose-6-phosphate to D-fructose-6-phosphate in glycolysis and gluconeogenesis, and facilitates the recycling of hexose-6-phosphate in the pentose phosphate pathway [14], [15]. The protein has an alpha/beta fold with an extensive dimer interface. The active site, identified based on sequence and structural alignments with related proteins, is formed at the dimer interface and comprises residues Arg270, Glu355 and His386, with the active site histidine donated from the partner monomer (Figure 6). The catalytic mechanism is an acid-base type mechanism with Glu355 acting as a putative general base, abstracting a proton from the substrate to facilitate the reversible isomerization of the substrate molecule. Histidine 386 donates a proton, facilitating the ring opening mechanism. As expected from the high sequence identity (over 60% identical), the *E. coli* pGI dimer aligns extremely well with the mammalian enzyme structures used for molecular replacement [15].

### 6-phospho-beta-glucosidase (BglA)

6-phospho-beta-glucosidase (BglA), is a cytoplasmic enzyme and part of the glycosal hydrolase family that is able to hydrolyse aromatic β-glucoside phosphates into glucose-6-phosphate and a hydroxyl aromatic *in vitro*. *E. coli* has several predicted enzymes in this family, whose physiological role has not been fully described. BglA forms an 8-strand alpha/beta TIM barrel with the putative catalytic residues Glu180 and Glu377 located in beta strands 4 and 7. Additional density was noted in the active site and a sulfate molecule from the crystallization conditions was well ordered adjacent to the putative catalytic residues Glu180 and Glu377 and formed hydrogen bonding interactions with Thr434 and Trp425. Modeling glucose-6-phosphate into the active site based on the position of the sulfate molecule revealed residues likely involved in substrate positioning (Figure 7).

### Glutamate dehydrogenase

Glutamate dehydrogenase (GDH), catalyzes the reversible oxidative deamination of glutamate to α-ketoglutarate and ammonia, using either NAD^+^ or NADP^+^ as a cofactor. The reverse reaction generates glutamate and the reduced cofactor NADH or NADPH, thus GDH has a pivotal role between carbon and nitrogen metabolism, particularly in plants and bacteria. The majority of characterized glutamate dehydrogenases are homo-oligomers, consisting of between two to six subunits, with dimers being the most frequent [16]. GDH from *E. coli* crystallized as a hexamer, consistent with biochemical observations [17], and contains two domains – a larger C-terminal Rossmann fold containing domain and a second N-terminal helical capping domain (Figure 8). The putative active site is located in a cleft between the two domains.

## Discussion

Current estimates suggest that *E. coli* cells produce over 1100 cytosolic soluble proteins that vary in abundance up to 5 logs [18]. Many of these proteins are expected to form macromolecular complexes, reducing the number of molecular species to less than 1000 [19], [20]. Our aim was to fractionate the crude lysate in several distinct ways in order to obtain as many of these species as possible in relatively pure fractions. Using a “macro-to-micro” approach that combined macro scale methods for the production and purification of native source proteins with novel microfluidic methods for protein sample analysis and crystallization, we were able to expand previous work that focused on proteome fractionation alone and to develop a robust structure determination pipeline using native source proteins.

Previous studies have shown that the soluble proteins of the *E. coli* proteome can be fractionated using ion exchange chromatography and unique proteins identified by mass spectrometry from 2-D gel spots [21]. Based on these studies that successfully simplified and fractionated the soluble portion of the proteome, we were able to scale-up these purification steps in order to obtain enough material for downstream crystallization experiments. The powerful combination of multiple chromatographic methods (tangential flow fractionation, ion exchange chromatography on high capacity resins, size exclusion chromatography, pH shifts, affinity chromatography, and limited proteolysis) to subdivide the *E. coli* proteome allowed the generation of unique protein samples for downstream crystallization and structure determination.

While in many cases lower purity samples may require further purification steps in order to be suitable for downstream crystallization experiments, it was observed that some proteins present in as little as 5% abundance still crystallized and yielded crystals of sufficient quality for structure determination (Figure 3 B and C). Thus, in contrast to the classical biochemistry approach where the final objective is to achieve a highly pure sample by removing trace impurities prior to crystallization, we found that many well diffracting crystals could be obtained from fairly complex samples. This successful crystallization from complex samples is most probably due to the inherent propensity of certain proteins to crystallize and will not be true in all cases. As this study sampled a broad swathe of the *E. coli* proteome, our results are likely biased towards proteins with a high degree of crystallizability.

Size-exclusion chromatographic characterization of the lower molecular weight protein fraction (less than 500 kDa) showed a maximum peak at approximately 100 kDa, while analysis of the ORF content of *E. coli* suggests a proteome size distribution peak at approximately 20 kDa (Figure 2 A and B). One possible reason for the size difference observed is the presence of a large number of homo- and hetero-oliogomers in the *E. coli* proteome. Studies of *E. coli* proteins and protein complexes demonstrated similar results, with the majority of proteins existing in complexes of varying stability [20]. In addition, all proteins crystallized were oligomeric. Oligomer formation has been postulated to increase the stability of a given protein species [22]. This likely selects for proteins that are highly stable and potentially more crystallizable [23].

Intracellular protein quantification studies of *E. coli* have shown that essential proteins are present with at least ten copies per cell and are present in higher concentrations than many non-essential proteins under steady state growth conditions [24]. We expected to see an over representation of high abundance and/or essential proteins in our crystallization experiments. Single cell protein concentration data were available for 14 of the 23 structures solved, and of these, only inorganic pyrophosphatase is an essential protein in *E. coli*. Nine of the non-essential proteins were high abundance (more than 10 copies per cell), while four of the remaining proteins had copy numbers ranging from 0.152 (pyruvate kinase I) to 2.6 (lysyl-tRNA synthetase) and are considered low abundance proteins. Thus, the structures determined did not sample only highly abundant proteins and/or essential proteins, but also sampled low abundance non-essential proteins. In addition, proteins involved in stress response such as methylglyoxal reductase (YghZ) and heat shock protein 31 (HSP31) [25] were crystallized. While these proteins are likely constitutively present at low levels under optimum growth conditions, the high cell density present during fermentor growth may lead to the induction of a stress response even when controlling for pH and aeration of the fermentor media. These results suggest a generally applicable method for altering the proteomic profile of a bacterial culture by manipulating the growth conditions to favor the production of proteins involved in specific pathways such as stress response. By boosting the protein concentration of specific proteins, the likelihood of their subsequent purification and crystallization can be increased.

A non-trivial issue was the identification of the proteins upon successful crystallization. The small quantities of available sample coupled with the relative complexity of the fractions made protein identification by mass spectrometry difficult. Studies using samples obtained from capillary gel electrophoresis couple with time of flight mass spectrometry have been shown to give good results with picogram quantities of material [26], [27]. It is likely that optimization of the mass spectrometric parameters and the use of equipment devoted to the identification of small amount of protein samples purified via capillary gel electrophoresis would greatly improve the success rate of mass spectrometry for routine protein identification. For this study, brute force molecular replacement provided a more robust method for the routine identification of crystallized protein samples, with a success rate of ∼80% for well diffracting (better than 3.5 Å) crystals.

Brute force molecular replacement (MR) was used to both identify the crystallized proteins and address the fundamental problem of phasing the crystallographic data. Native data does not allow the *ab initio* structure determination and either MR search models, heavy atom derivatives, or, in more limited cases, changes due to radiation damage, are needed to successful phase a structure. In the case of inorganic pyrophosphatase, for example, the crystals were isomorphous to published structures in the Protein Data Bank (PDB) and hence a strong candidate was identified based on the cell constants and symmetry information alone. For the majority of the crystal structures, cell constants were not sufficient to identify the protein, leading to the use of brute force MR trials. The relatively simple MR protocol used in this study was designed to run on limited computational resources (typically 1 week on a single-processor system), which reduces the search space and sensitivity. Recent advances in the application of structure prediction methods [28], [29], [30], [31] and assembly of helical fragments [32] have expanded the range of structures accessible by MR to the point where it resembles true *ab initio* phasing, at the cost of massive computational overhead [33]. However, the ongoing evolution of multiprocessor systems, and the availability of massively parallel computing “grids” [34], will make these technologies more accessible to the average laboratory.

The twenty-three structures determined here represent a significant fraction of the 767 unique structures deposited in the PDB for *E. coli,* one of the most structurally studied model organisms. Because the PDB contains over 75,000 structures and an increasing amount of “fold space” is being sampled due in large part to the efforts of the structural genomics consortia [35], brute force molecular replacement is fast becoming an increasingly viable method for structure solution [34], [36], [37]. However, MR was not sufficient to determine six structures for which we collected native data of resolution better than 3.5 Å. Although our sample size is small, our 40% success rate (4 solved/10 collected of resolution better than 3.5 Å) with novel structures using MR suggests that significantly more structures would need to be solved to completely access the meta-proteome using MR strategies alone.

In addition to MR, attempts were made at heavy atom derivatization using mercury, NaBr and NaI soaks as well as phasing using the sulphur edge. While these approaches were successful on test crystals, we were not able to obtain useful phase information for the remaining unsolved data sets. Heavy atom derivatization often requires extensive optimization of soaking conditions through trial and error and thus a large number of crystals. As we were limited in the number of crystals available and the amount of sample for crystal optimization, this approach was not successful. Generally our crystals were small and suffered from radiation damage after collection of a complete dataset. The highly redundant data necessary for sulphur SAD phasing was not obtainable for the unsolved data sets. However, optimization of data collection parameters such as wavelength and exposure times would likely improve the success rate of sulphur SAD. Radiation induced phasing (RIP) [38], [39] was not attempted in the current study but is another alternative method to use in order to solve the phase problem. For bacterial targets such as *E. coli*, selenomethionine incorporation provides an alternative to traditional heavy atom derivitization and works routinely for recombinantly expressed proteins. A drawback to this approach is the toxicity and cost of selenomethionine. An estimated 500 g of *E. coli* grown in a 120 L fermentor would require at least twelve grams of L-selenomethionine, a substantial cost and disposal issue. However, for some bacterial targets this may provide an attractive alternative to relying on MR solutions or heavy atom soaking.

These studies comprise a first attempt to explore the feasibility and potential of using a macro-to-micro approach to fractionate and purify proteins exclusively from a native source for high-throughput crystallization and structure determination. As the scope of this pilot study is relatively small, the aim was to test an initial production pipeline using the model organism, *E. coli*. The success and efficacy of our native-source purification and crystallization were assessed by comparing the number of datasets collected compared with the number of available structures in the PDB and also the number of unknown protein structures identified and solved using native-source purification. The general utility of this approach is not limited to bacteria, but can be easily adapted to structurally study the proteomes of higher organisms or proteomes of specific tissues during different developmental stages, provided that a sufficient starting pool of protein is available for fractionation, purification, and crystallization. Not only is the pipeline presented here applicable to different source organisms or specific tissue types, but it is also feasible on a single lab scale. The combination of native source protein purification with novel microfluidic technology enables crystallographic characterization of protein samples orders of magnitude smaller than traditional crystallographic methodologies would suggest. These results validate the “macro-to-micro” approach as a complementary method to recombinant methodologies currently employed by structural genomics initiatives.

## Methods

### Preparation of Soluble Protein Extracts from *E. coli*

A 4 L culture of *E. coli* DH5α cells grown overnight in minimal media by shaking at 37°C was used to inoculate a 120 L fermentor containing minimal media. The cells were grown to log phase, harvested by continuous-flow centrifugation, washed, and frozen in liquid nitrogen. The 1 kg cell pellet was resuspended in 3 L of lysis buffer (10 mM NaCl, 25 mM Tris pH 7.0, 1 mM DTT and 0.05% Triton X-100) and lysed by two passages through an EmulsiFlex-C3 homogenizer at 10,000 psi. The cell debris was removed by centrifugation for 60 min at 15,000 rpm. A Bradford protein assay was used to determine the protein concentration using Bio-Rad Protein Assay Dye. The lysate was treated with protease inhibitors and the nucleic acids were digested using DNaseI.

### Whole-proteome fractionation pipeline

Tangential flow filtration (TFF) using the ProFlux M12 (Millipore) with a nominal molecular weight cutoff (NMWC) of >500,000 Da was used to remove cell debris, aggregates, and large complexes from the lysate. The retentate was purified using a sequence of three purification steps: sucrose gradient→TSK-Gel G4000SW→ion exchange chromatography on an 8 ml MonoQ column (GE Healthcare Life Sciences). The permeate, at <500,000 Da, was subjected to a sequence of three or more orthogonal purification steps (affinity interactions, multiple ion exchangers, hydrophobic interaction and/or size exclusion). In a standard protocol, 50 to 500 ml of the permeate was applied to different capture resins, with column volume and permeate volume scaled according to the capacity of the capture resin. The initial separation step was performed on one of the five choices of resins. The capture columns used were CaptoQ columns (quaternary ammonium, anion exchanger resin), Capto MMC (multimodal weak cation exchanger resin), Blue Sepharose columns (triazine coupling resin), Heparin Sepharose columns (reductive amination resin) or on SP Sepharose columns (Sulfopropyl strong cation resin). The columns were eluted in 3 to 5 steps, and each subsequent step was then applied to a Phenyl sepharose column and further subdivided into 6 fractions. These fractions were then applied to a preparatory scale Superdex 200 column (GE Healthcare Life Sciences) or a polishing high resolution ionic exchange 8 ml MonoQ or MonoS column (polystyrene/divinyl benzene particles substituted with quaternary amino (Q) or methyl sulfonate groups (S). The eluted fractions were 1 ml in volume and collected in 96 well trays. For example, YghZ was purified via heparin sepharose, phenyl sepharose, and then a polishing MonoQ step. BglA, GDH, and pGI were purified in a similar manner as YghZ, with the exception of the initial heparin column being replaced by a CaptoQ step.

The purity of the fractions was assessed by 1-D electrophoretic separation with the LabChip90 Caliper LifeSciences System. Similar fractions were pooled and concentrated using spin concentrators to 10–20 mg/ml for crystallization. Mass spectrometry was used for the identification of the fraction of samples that crystallized.

### Mass spectrometry

Mass spectrometry and tandem mass spectrometry (MS/MS) were performed using a quadrupole time-of-flight mass spectrometer (Q-tof Premier, Waters, Milford, MA) that was equipped with a nanoelectrospray ionization source and connected in-line with an ultraperformance liquid chromatograph (nanoAcquity UPLC, Waters). ESI mass spectra of intact proteins were processed using MassLynx software (version 4.1, Waters). The data resulting from UPLC-MS/MS analysis of trypsin-digested proteins were searched against the Swiss-Prot database using ProteinLynx Global Server software (Waters). Protein identifications were validated by manual inspection of the MS/MS spectra.

### Crystallization

All the fractions were screened in Topaz™ 8.96 (Fluidigm Corporation) microfluidic crystallization chips against Index and OptiMix screens at room temperature, using approximately 1 µL of protein sample per 96-well screen. The results were viewed and analyzed by the AutoInspex® station, which records images of each experiment over a 7 day period and automatically scores each experiment. Samples yielding high-quality crystals were reproduced and optimized in Greiner 96 well sitting drop plates using the Mosquito® crystallization robot (TTP Labtech). Depending on amount of sample available, optimization conditions were chosen to sample a range of precipitant conditions, generally +/−20% from the initial crystal hit. Lower quality crystals were improved by screening crystallization additives or further sample purification. For small sample volume and in cases where crystals from the initial screen were not reproduced by vapor diffusion, diffraction-capable microfluidic chips (Fluidigm Corp.) were screened for diffraction.

### Data Collection

Crystals grown in conventional trays were cryoprotected prior to flash freezing in liquid nitrogen by transferring to a solution containing either a higher (>30%) concentration of precipitant where possible (PEG 1000, 3500-based conditions) or 50% glycerol. Crystals grown in diffraction-capable chips were cryoprotected by cutting open liquid channels and allowing a 50% glycerol solution to diffuse into the crystallization chamber. Sections of the chips containing crystals were then excised and attached to magnetic mounting pins before freezing. Data were collected at ALS beamlines 8.3.1, 12.3.1, and 8.2.1, and SSRL beamlines 9-1 and 9-2, and processed with HKL2000 [40],XDS [41], or MOSFLM [42] and SCALA [43], using the Elves automation software [44]. All data sets were obtained from a single crystal except for the in-chip crystals of 5-keto-4-deoxyuronate.

### Structure Determination and Refinement

The space group and unit cell dimensions of each crystal were used to search the *E. coli* proteins in the PDB for candidate molecules. Mass spectrometry was used to determine the identity of unknown crystallized proteins, where possible. In all other cases, brute-force molecular replacement (MR) trials were conducted using all structures in the PDB with at least 30% sequence identity to an *Escherichia coli* ORF. At the time of these experiments, there were approximately 10,000 unique structures with at least 30% sequence identity to an *E. coli* open reading frame. Decreasing the percent sequence identity to 25% yielded approximately 15,000 structures, however, no further MR solutions were found for the datasets. The program MOLREP [8] was used to calculate scores for the rotation function alone, the highest scoring functions were selected, and the candidate structures were subjected to full MR using MOLREP or PHASER [9]. In the case of YghZ, MR alone could not distinguish between several homologous ORFs, and the model was partially refined and rebuilt with ARP/wARP [45]–[46]to an R-free of 37%, at which point the high resolution of the data allowed identification of unique sequences in the electron density.

Structures for proteins already deposited in the PDB were not refined except to confirm the molecular replacement solutions. For the novel structures, initial models were refined using either REFMAC [43], [47] or phenix.refine [48], [49], and rebuilt using ARP/wARP or phenix.autobuild [50] where the resolution was high enough. The rebuilt models were then iteratively refined with manual building in Coot [51] and refinement with phenix.refine or REFMAC. NCS restraints were applied throughout refinement until the final stages, where they were released if resolution was 2.1 Å or better. PyMOL [52] was used to generate all structure figures. Novel structures are deposited in the PDB under codes 3N6Q, 2XHY, 3NBU, 3SBO for YghZ, BglA, pGI, and GDH, respectively. Ramachandran statistics for YghZ were 97% (favored), 2.6% (allowed), 0.4% (disallowed), for BglA 97.1% (favored), 2.8% (allowed), 0.1% (disallowed), for pGI 96% (favored), 3.8% (allowed), 0.3% (disallowed), and for GDH 86.1% (favored), 13.5% (allowed), 0.4% (disallowed).
